# Increasing Mentalization to Reduce Maladaptive Defense in Patients With Mental Disorders

**DOI:** 10.3389/fpsyt.2021.637915

**Published:** 2021-03-11

**Authors:** Markus C. Hayden, Pia K. Müllauer, Klea J. P. Beyer, Richard Gaugeler, Birgit Senft, Maria C. Dehoust, Sylke Andreas

**Affiliations:** ^1^Institut für Psychologie, Alpen-Adria-Universität Klagenfurt, Klagenfurt, Austria; ^2^Klinik Bad Reichenhall der Deutschen Rentenversicherung Bayern-Süd, Bad Reichenhall, Germany; ^3^Öffentliches Krankenhaus Waiern, Feldkirchen in Kärnten, Austria; ^4^Reha-Klinik für Seelische Gesundheit und Prävention, Klagenfurt, Austria; ^5^Psychosomatische Klinik Ginsterhof, Rosengarten, Germany

**Keywords:** mentalization, psychological defense mechanisms, maladaptive defense, psychopathology, interpersonal problems

## Abstract

**Background:** There are indications of associations between the ability to mentalize and psychological defense mechanisms. However, only a few studies have focused on these associations, and even fewer have included empirical analyses. In the present study, we aimed to fill this research gap by analyzing the link between the ability to mentalize and psychological defense mechanisms in patients with mental disorders. We examined whether changes in defense mechanisms are predicted by an increase in mentalization or whether such changes are only related to reductions in psychopathology and interpersonal problems.

**Methods:** A clinical sample of *N* = 89 patients was studied during and after inpatient psychiatric rehabilitation. Repeated-measures analyses of variance were performed to determine changes in mentalization, psychological defense, psychopathology, and interpersonal problems over the course of therapy and post-treatment. Linear regression analyses were used to predict the change in defense patterns based on an increase in mentalization.

**Results:** Maladaptive defense mechanisms were significantly reduced during inpatient therapy and remained low until follow-up, whereas neurotic and adaptive defense mechanisms did not change significantly. The results of the regression analyses indicated that mentalization played an important role in the reduction in maladaptive defense during and after inpatient rehabilitation for mental disorders, whereas reductions in psychopathology and interpersonal distress were only partially associated with a reduction in maladaptive defense.

**Conclusion:** We conclude that mentalization is vital for reducing maladaptive defense mechanisms, which are commonly associated with mental disorders. In therapy, an increase in patients' capacity to mentalize may be a practicable approach to diminish maladaptive defense mechanisms.

## Introduction

Mentalization is a form of mostly preconscious imaginative mental activity that is defined by the ability to understand and interpret one's own and others' behavior in terms of underlying mental states. These states go beyond thoughts, feelings, and emotions and include needs, beliefs, goals, purposes, and reasons (1–3). High levels of mentalization are characterized by a differentiated understanding of the inner world that affects human beings and include the awareness that the mind, especially the mind of another person, cannot fully be accessed or read (2). Mentalizing enables humans to reflect upon their own and other people's perceptions and understand and anticipate associated patterns of behavior. Therefore, it plays a key role in interpersonal behavior (4, 5). Furthermore, it has proven to be a substantial factor influencing the transfer of attachment security from parents to their children (6). Mentalization is a broad concept that encompasses aspects of the self vs. others as well as both implicit and explicit and both cognitive and affective dimensions (3). Therefore, there are several conceptual overlaps between mentalization and other models, such as mindfulness, empathy, affect consciousness, and theory of mind (3, 7). The most common way to operationalize and measure the capacity to mentalize is through reflective functioning (8). To date, various instruments, such as interviews and questionnaires, have been developed for the assessment of reflective functioning (9). Mentalization is positively intercorrelated with mental stability and attachment security. A growing number of studies have emphasized the importance of mentalization as a protective factor against mental disorders (10–12). On the other hand, impairments in the ability to mentalize are predictors of psychopathology and mental instability (3, 8). In the last decade, several studies have clarified the associations between a lack of mentalization and various kinds of mental disorders e.g. (13–16). In particular, mental disorders that involve a pathology of the self, such as borderline personality disorder, are characterized by a distinct pattern of impairments in the ability to mentalize (3, 8).

In contrast to the relatively new field of mentalization research, studies on psychological defense have been conducted since the late 19th century (17). Sigmund Freud published his first work on defense in 1894 (18) and continued his research in the field for several decades e.g. (19–21). His studies and the research published by his daughter, Anna Freud (22), described the main characteristics of psychological defense and most of the defense styles that are known today (23). Psychological defense mechanisms are characterized as unconscious mental processes that provide important self-protective effects by reducing or masking anxiety arising from unacceptable or potentially harmful stimuli (24, 25). In particular, defense mechanisms maintain psychological homeostasis, i.e., the organization of personality, in both pathological and healthy individuals (25, 26). In the absence of defense mechanisms, humans are persistently confronted with negative emotions, such as anger, sadness, and anxiety (24). Defense mechanisms are vital for a healthy relationship with the self, others, and the environment. However, these mechanisms have the ability to be potentially harmful as well, depending on the manner, frequency, and circumstances in which they are unconsciously used (27). Various forms of defense styles evolve from infancy to adolescence and adulthood, making the individual more flexible in defending himself or herself against negative stimuli (22). Contemporary psychology has adapted a hierarchical understanding of different forms of defense mechanisms based on their level of adaptiveness (28). Healthy individuals can draw on a variety of defense mechanisms that match the circumstances in which they are used. People with mental disorders, however, tend to use only a limited range of defense mechanisms that may not be adapted to the situation, for example, with respect to the individual's age or the duration or intensity of the stimulus (22). In particular, immature (or maladaptive) defense styles are frequently used by patients suffering from mental disorders (29). Furthermore, research has revealed links between physical impairment and the use of different forms of defense styles. For example, studies found that the use of immature defense mechanisms may be associated with somatic symptom severity (30) and may contribute to impaired awareness in patients with traumatic brain injury (31). Other studies identified the role of psychological defense mechanisms in patients with cancer [see (27) for a review]. Defense mechanisms are known to be relatively stable in adulthood; however, they are well documented to be dynamic and reversible, e.g., via psychotherapy (28, 32, 33).

There are several indications of associations between mentalization and defense mechanisms. For example, both mentalization and defense mechanisms play important roles in the preservation of mental stability, whereas impairments are linked to psychological strain and mental disorder. Associations have also been reported in clinical research, for example, in patients suffering from alexithymia and borderline personality disorder. Both disorders are characterized by a lack of mentalization, and both disorders are characterized by the predominance of immature defense mechanisms (34, 35). Furthermore, both the enhancement of reflective functioning and the maturation of defense mechanisms are associated with mental stability and with progress in psychotherapy, e.g., in the treatment of personality disorders (11, 28, 36, 37). Since mentalization enables humans to reflect upon their own actions and, in particular, to reflect upon the mental processes that cause their own actions, an association of mentalization with the use of various kinds of defense mechanisms seems plausible. There are also indications that an increase in mentalization, as measured by reflective functioning, may enable individuals to scrutinize their own defense mechanisms, which can in turn increase their overall capability to mentalize (38).

However, there is hardly any detailed research on the associations between mentalization and the use of psychological defense styles. Only isolated studies have considered these possible associations, and even fewer studies have empirically investigated the possible intercorrelations. Shahar and colleagues (39) emphasized a possible link between impairments in mentalizing capacities and the use of immature defense mechanisms, such as projection. The authors stated that individuals with lower mentalization scores were restricted in their use of defense mechanisms, as they had struggles identifying their own mental states and those of others. This may be an explanation for why highly burdened individuals have a tendency to use immature defense mechanisms, as people are less likely to tap the full potential of their own mentalizing abilities in situations of high emotional burden (39). In one case study (40), the author presented a phobic patient who continuously used the inhibition of mentalization as a defense against mental threats. Finally, in a study by Fischer-Kern and colleagues (35), the correlations between the primitive defenses dimension of the Structured Interview of Personality Organization (41) and reflective functioning were calculated for a sample of *N* = 92 female outpatients with borderline personality disorder. The analysis did not find significant intercorrelations. However, the reflective functioning scores were very homogenous, with the means of the dimensions ranging from 2.4 (SD 1.1) to 2.9 (SD 1.5) (35).

Similar to the associations between psychological defense and mentalization, the associations between defense styles and concepts related to mentalization have hardly been studied. One study that analyzed *N* = 107 students and graduates detected positive intercorrelations between the use of adaptive defense styles and both emotional knowledge and overall emotional intelligence as well as a negative correlation between maladaptive defense styles and emotional knowledge (42). Furthermore, Brown and colleagues (43, 44) pointed out that mindfulness can lead to less ego-defensive responsivity under social threat. In line with their assumptions, one study comparing an intervention group (*N* = 438) with *N* = 281 controls found that a seven-day Vipassana meditation retreat, as an intervention to foster mindfulness, led to a reduction in the use of immature defense mechanisms, namely, displacement, regression, and projection (45).

Since the relationship between the ability to mentalize and psychological defense has not been studied in a structured way, and there is hardly any empirical research apart from some scattered results, a link between the two concepts can currently only be hypothesized. Furthermore, it is unclear whether changes in the capacity to mentalize are linked to changes in the use of defense mechanisms. Therefore, the present study analyzed patterns of associations between mentalization and the use of different psychological defense mechanisms. Because both variables are known to be affectable by treatment (11, 28), we investigated the potential relationship in patients with mental disorders over the course of inpatient therapy and during the posttreatment follow-up. The focus of the study was on patients' subjective experiences as measured by patient-reported outcomes (46). First, we analyzed the degree to which the investigated variables changed over the course of therapy and follow-up. Then, we sought to determine which variables predicted changes in defense mechanisms. We hypothesized that these changes would be predicted not only by reductions in debilitating mental factors, i.e., psychopathology and interpersonal problems, but also by an increase in mentalization.

## Materials and Methods

### Design

The study was designed as a quasiexperimental longitudinal study. We surveyed patients at the beginning of inpatient therapy for psychiatric disorders (T_0_) and shortly before discharge from the hospital (T_1_). Furthermore, a follow-up measurement was conducted approximately half a year later (T_2_).

### Instruments

We used the 40-item German version of the Defense Style Questionnaire [DSQ-40 (47)] to analyze psychological defense mechanisms. The self-report instrument is a shortened version of the Defense Style Questionnaire presented by Andrews, Pollock, and Stewart (48), and it has widely been used and studied. The DSQ-40 has been translated into various languages and has proven to be suitable in both adult and adolescent populations (26). The instrument has three dimensions that were used in the analyses: adaptive defense, intermediate (neurotic) defense, and maladaptive defense. Cronbach's alphas range from 0.58 to 0.80. The test–retest coefficients range from 0.75 to 0.85 (47). To cluster the variables according to these main categories, we followed the recommendations of Schauenburg et al. (49), describing minor adaptions to the German version compared to the original version.

To assess the ability to mentalize, we used the global scale of the German version of the Mentalization Questionnaire [MZQ (50)]. This 15-item self-report instrument has proven to be a reliable and valid tool in the assessment of mentalization and yields results that are comparable to those generated by interview measures, such as the Adult Attachment Interview (Andreas et al., submitted). Several translated versions of the MZQ have been used in adult and adolescent populations (50–52). For the original German version, Cronbach's alpha for the global scale is 0.81 and the test-retest reliability is 0.76 (50).

Psychopathology was assessed using the Global Severity Index (GSI) of the German version of the Brief Symptom Inventory 18 [BSI-18 (53)]. The instrument is the latest short version of the Symptom-Checklist 90-R. A study that included *N* = 2516 participants demonstrated the psychometric qualities of the German version (54). The GSI score represents the number and severity of the psychopathological symptoms assessed by the BSI-18. Cronbach's alpha for the GSI of the German version is 0.93 (54).

The German 32-item version of the Inventory of Interpersonal Problems [IIP-32 (55)] was used to assess difficulties within interpersonal contact. The questionnaire asks patients to rate items concerning actions (e.g., in groups or other forms of interpersonal contact) that they “do too much” and that they find “too hard to do” (56). A study by Thomas et al. (55) demonstrated that the quality indicators of the German version of the IIP-32 are comparable to the original version of the IIP. Values for Cronbach's alpha range from 0.60 to 0.82 in a standard population and from 0.59 to 0.83 in clinical populations (55). For our analyses, we used the full scale that represents the total amount of distress experienced in interpersonal contexts.

### Data Collection

The data were collected in two hospitals in Austria that offer psychiatric rehabilitation. In addition to medical and pharmacological treatment options, both hospitals use psychotherapy in one-on-one settings as well as group interventions. The therapy plans further include psychoeducation, ergotherapy, physiotherapy, and physical exercise. All patients in both hospitals were at least 18 years old. The standard duration of treatment at the hospitals ranges from three to six weeks. At the beginning of therapy, all patients are diagnosed according to the ICD-10 (57).

Participation in the study was voluntary, and all the patients were informed that neither their refusal to participate nor their later withdrawal from the study would have any consequences whatsoever, particularly regarding therapy and aftercare. The exclusion criteria were an inability to complete the study questionnaire and/or take part in diagnostic interviews (i.e., an insufficient ability to understand and/or speak German, acute manic or psychotic episodes, dementia, or other forms of cognitive impairment). The study was approved by the ethical commission in charge. All patients who did not meet the exclusion criteria were asked to take part in the study within the first 4 days of therapy. For the follow-up assessments, all the patients were contacted via telephone. If a participant could not be reached, we sent a standardized form in the mail to contact them.

### Data Analysis

The data were analyzed using IBM SPSS Statistics 25. The analysis of missing values showed a missing rate of >5% at both the case and variable levels. In total, ~12.3% percent of the data were missing. Little's test of missing completely at random (58) was not significant (chi-squared = 308.533, df = 347, *p* = 0.932), indicating that the data were missing completely at random. For the replacement of missing values, we used multiple imputation to obtain a complete dataset. In accordance with the recommendations of White, Royston, and Wood (59), we calculated twelve imputations.

To check for possible differences between the subsamples, we calculated an independent samples *t*-test for age and chi-square tests for all the other sociodemographic variables. For the *t*-test, homogeneity of variances was tested using Levene's test for equality of variances (60).

Repeated-measures analyses of variance (rmANOVAs) were used to analyze the changes in the variables over the course of the therapy and post-treatment. The exploratory data analysis indicated that there were no outliers in the data. Mauchly's sphericity test was used to detect violations of sphericity. Since all violations that could be detected were at the level of ε > 0.75, the analyses were adjusted using the Huynh-Feldt procedure (61).

For the main part of the study, we used linear regression analyses to predict a decrease in maladaptive defense. The decrease was calculated by subtracting the T_1_ values from the T_0_ values to determine the changes over the course of therapy and by subtracting the T_2_ values from the T_0_ values to determine the difference between the baseline assessments and the follow-up assessments. Changes in interpersonal problems and symptom severity were calculated in the same way. Since mentalization was reverse coded, an improvement was expressed as an increase in the MZQ score. Therefore, we subtracted the T_0_ values from the T_1_ values to determine changes during therapy and the T_0_ values from the T_2_ values to determine the difference between the baseline assessments and the follow-up assessments. There were no indications of multicollinearity (maximum variance inflation factor = 1.829) or autocorrelation (Durbin-Watson statistics = 1.744 and 2.078, respectively). Controlling the scatterplot did not reveal any indications of heteroscedasticity. Shapiro-Wilk tests of studentized residuals did not reach statistical significance (*p* = 0.493 for T_1_ and *p* = 0.113 for T_2_), suggesting a normal distribution of the residuals in both analyses. Casewise diagnostics indicated one case in the first analysis and two cases in the second analysis as outliers on the y-axis. However, neither of these values had a leverage above the cutoff of 2*k*/*n* that would also indicate extreme x-values (62). In all three cases, the values for Cook's distance (63) were below the cutoff of ≥1, which would indicate a problematic influence on the analyses.

## Results

### Participants and Dropouts

Eighty-nine patients were willing to take part in the study. The sociodemographic parameters of the sample are displayed in Table 1. The majority of the participants (*n* = 61, 68.5%) had main diagnoses on the F3 spectrum according to the ICD-10 (57), followed by those with F4 diagnoses. Fifty-four participants (60.7%) had more than one diagnosis. Further information on the distribution of the diagnoses is displayed in Table 2. An analysis of the differences between the two subsamples is included below.

**Table 1:** Sociodemographic variables of the clinical sample.

Age	M	44.0
	SD	9.79
	Range	22–63 years
Sex	Male	42 (47.2%)
	Female	47 (52.8%)
Civil status	Single	26 (29.2%)
	Living in Partnership	16 (18.0%)
	Married	28 (31.5%)
	Divorced or widowed	19 (21.3%)
Children	Yes	56 (62.9%)
	No	33 (37.1%)
Education	Elementary	1 (1.1%)
	Main School	25 (28.1%)
	Professional School	15 (16.9%)
	High School	17 (19.1%)
	University	14 (15.7%)
	Other	17 (19.1%)

**Table 2:** Distribution of diagnoses as absolute frequencies.

**Diagnosis according to the ICD-10**	***n* main diagnosis**	***n* 2nd diagnosis**	***n* 3rd diagnosis**
F1* Mental and behavioral disorders due to psychoactive substance use	0	13	11
F2* Schizophrenia, schizotypal and delusional disorders	3	0	0
F3* Mood disorders	61	6	1
F4* Neurotic, stress-related and somatoform disorders	24	23	3
F5* Behavioral syndromes associated with physiological disturbances and physical factors	0	3	3
F6* Disorders of adult personality and behavior	0	4	1
F9* Unspecified mental disorder	0	1	0
Other diagnoses/not F-diagnoses	1	4	1
**SUM**	**89**	**54**	**20**

Between T_0_ and T_1_, *n*=3 patients (3.4%) dropped out of the study; two participants quit because they had no further interest in the study, and one participant had to be excluded from the study because of an acute psychosocial crisis. Between T_1_ and T_2_, another *n* = 15 patients (16.9%) dropped out of the study. The most common reason for dropout (*n* = 9, 10.1%) was that patients could not be reached at follow-up. Two participants had no further interest in the study, one participant quit because of an acute physical disease, one participant was deceased, one participant did not specify the reason for withdrawal from the study, and one participant had to be excluded from the study because of a labile mental status.

### Differences Between the Subsamples

There were no statistically significant differences between the subsamples in age (*p* = 0.840), sex (*p* = 0.353), employment status (*p* = 0.056), level of education (*p* = 0.114), parenthood (*p* = 0.951), or the distribution of diagnoses (*p* = 0.269). The only significant difference that was found was in civil status (*p* = 0.035), with the patients from one hospital being more likely to report a single civil status at T_0_.

### Changes Over the Course of Therapy and During Follow-Up

The rmANOVAs indicated that among the three dimensions of the DSQ-40 (49), neither adaptive defense nor intermediate (neurotic) defense significantly changed over time (see Table 3). However, maladaptive defense was significantly reduced. A *post hoc* analysis revealed that the patients reported significantly fewer maladaptive behaviors at T_1_ than at T_0_. At follow-up, the use of maladaptive defense mechanisms was reduced even further, but the difference between T_1_ and T_2_ did not reach statistical significance. All the other variables significantly improved over the course of therapy and post-treatment.

**Table 3:** Results of the rmANOVAs.

	***F*-statistics**	**Significance**	**Partial η^**2**^**
DSQ adaptive defense	*F* (1.872, 164.756) = 1.023	*p* = 0.358	0.011
DSQ intermediate defense	*F* (1.892, 166.487) = 2.221	*p* = 0.115	0.025
DSQ maladaptive defense	*F* (2, 176) = 10.228	*p* < 0.001	**0.104**
MZQ global scale	*F* (2, 176) = 11.355	*p* < 0.001	**0.114**
GSI	*F* (1.737, 152.862) = 39.554	*p* < 0.001	**0.310**
IIP full scale	*F* (2, 176) = 7.565	*p* = 0.001	**0.079**

*DSQ, Defense Style Questionnaire 40; GSI, Global Severity Index; IIP, Inventory of Interpersonal Problems 32; MZQ, Mentalization Questionnaire. The bold values indicate significant p-values*.

Since the changes over the course of inpatient therapy and post-treatment in adaptive and intermediate defense styles did not reach significance, these two variables were excluded from the subsequent analyses.

### Prediction of a Decrease in Maladaptive Defense

In the final step of the analyses, we investigated whether a decrease in maladaptive defense mechanisms could be predicted by an increase in mentalization or whether the decrease would be explained only by a reduction in psychopathology and/or interpersonal problems. Therefore, two linear regression models were calculated for the duration of treatment and for the period from admission to hospital until follow-up. Because there was no significant change between T_1_ and T_2_, this analysis was not conducted. The results of the analyses are displayed in Table 4.

**Table 4:** Results of the linear regression analyses.

**Reduction in maladaptive defense T_0_ to T_1_**		
*R*^2^ = 0.176, adjusted *R*^2^ = 0.146, *F*(3, 85) = 6.034, *p* = 0.001		
Increase in mentalization	Standardized β = 0.280	*p* = 0.008
Reduction in psychopathology	Standardized β = 0.021	*p* = 0.845
Reduction in interpersonal problems	Standardized β = 0.252	*p* = 0.019
**Reduction in maladaptive defense T_0_ to T_2_**		
*R*^2^ = 0.297, adjusted *R*^2^ = 0.272, *F*(3, 85) = 11.960, *p* < 0.001		
Increase in mentalization	Standardized β = 0.387	*p* = 0.002
Reduction in psychopathology	Standardized β = 0.204	*p* = 0.047
Reduction in interpersonal problems	Standardized β = 0.054	*p* = 0.641

As shown, we obtained two significant models. The *R*^2^ for the first model was 0.176 (adjusted *R*^2^ = 0.146), indicating moderate goodness of fit according to Cohen (64). The reduction in maladaptive defense was significantly predicted by both an increase in mentalization and a reduction in interpersonal problems.

For the second model, the *R*^2^ of 0.297 (adjusted *R*^2^ = 0.272) indicated high goodness of fit (64). The increase in mentalization was again found to be a significant predictor of a decrease in maladaptive defense. However, between T_0_ and T_2_, a reduction in psychopathology was also a significant predictor, whereas interpersonal problems did not significantly affect the data.

In both analyses, an increase in mentalization that was observed over the course of the inpatient therapy and the posttreatment period significantly predicted a reduction in maladaptive defense. On the other hand, a reduction in psychopathology as well as a reduction in interpersonal problems were not found to be persistent predictors of a reduction in maladaptive defense. Both variables predicted a decrease in maladaptive defense at one measurement timepoint only.

## Discussion

The aim of the study was to analyze the associations between the ability to mentalize and psychological defense mechanisms in a clinical sample. To the best of our knowledge, this is the first study to investigate the role of mentalization in changes in defense mechanisms during and after inpatient treatment for mental disorders.

As expected, the participants significantly improved over the course of inpatient therapy. Values for interpersonal distress decreased with a medium effect size, whereas psychopathology decreased with a large effect (64). In addition, mentalization could be significantly targeted, and the patients' mentalization scores increased over the course of therapy. The results remained stable until follow-up, indicating that the treatment had continuing effects on the patients' mental well-being. The outcomes for these three variables are consistent with a variety of previous results and confirm that inpatient therapy promotes mental health, with the promoting effect persisting after discharge from the hospital [e.g., (11, 13, 65, 66)]. Regarding psychological defense, maladaptive defense mechanisms decreased with a medium effect size during treatment and remained stable throughout follow-up. Adaptive and intermediate defense styles, however, did not change significantly over the course of inpatient therapy and post-treatment. In the comparison of these outcomes with previous studies, it is salient that some authors have reported similar results (67, 68), whereas others have reported significant improvements in more mature defense styles via therapy (28, 69, 70). A more comprehensive evaluation of previous investigations reveals that changes in more mature defense styles are linked to treatment with a long duration, which was not implemented in the current study. Therefore, longer inpatient therapy or structured ambulatory aftercare may have led to significant improvements in the patients' intermediate and/or adaptive defense styles.

Since adaptive and intermediate defense styles could not be targeted during the therapy, we excluded these two variables in the analyses and focused on the reduction in maladaptive defense mechanisms. According to our data, a decrease in maladaptive defense was more closely associated with an increase in mentalization rather than a reduction in interpersonal distress or symptom severity. While the latter two variables significantly predicted a reduction in maladaptive defense at one measurement period only, mentalization was found to be a significant predictor both between the beginning and the end of therapy as well as between the beginning of therapy and follow-up. This finding indicates that a more reflective view on one's own and perhaps other people's mental states, which supports the enhancement of mentalization, can enable patients to overcome hindering, immature defensive behavior. This outcome contrasts with the results of Fischer-Kern et al. (35), who did not find a significant correlation between mentalization, as measured by reflective functioning, and the use of primitive defense styles. However, in light of previous research, the associations found in our investigations can still be considered plausible given that mentalization plays a key role in determining and controlling emotions (2) and that humans who use more rigid emotional regulation strategies are prone to maladaptive defense styles (71, 72).

Importantly, the reduction in maladaptive defense mechanisms did not accompany a significant increase in adaptive or intermediate defense mechanisms. In other words, even though maladaptive defense styles were less commonly used by the patients, we could not detect a more frequent use of more mature defense mechanisms. This finding suggests that mentalization can help patients adopt healthier ways to cope with stressful stimuli by overcoming debilitating defense styles but not by increasing their use of mature defense styles.

While it is important to interpret the results considering the associated limitations, some implications can be identified based on the comprehensive literature on the effect of defense mechanisms on quality of life (22–24). In the treatment of patients with dominant maladaptive defense styles, the promotion of mentalization may be a practicable approach for therapy progress. This strategy seems particularly appropriate if alternative treatment options have failed to produce the intended results. In general, our results support the advice to implement interventions that target an increase in the capacity to mentalize. In addition to the well-documented advantages for psychotherapy e.g. (5, 8, 11, 13, 73, 74), our results suggest that through an increase in mentalization, patients may adapt a healthier overall defense style by reducing maladaptive elements. Since numerous studies have highlighted the importance of well-performing psychological defense in the rehabilitation and preservation of mental health (22, 24, 28, 29) as well as on humans' ability to adapt to and cope with severe medical conditions (27, 30, 31) and other forms of traumatizing life events (24, 75, 76), we consider interventions to foster mentalization as necessary in the reduction of maladaptive defense styles in psychotherapy. Regarding future research on psychological defense, our results suggest taking mentalization into account. This is particularly advisable when changes in defense styles are studied in the context of psychiatric or psychotherapy research.

### Strengths and Limitations

The study combines two factors that are of particular importance in mental health, namely mentalization and psychological defense, and it is the first to empirically investigate the interaction between these factors in patients recovering from mental disorders. Further strengths are linked to the longitudinal design. First, we were able to detect and analyze changes in the main variables over the course of inpatient treatment and further after the discharge from the clinic. Second, the study design also allowed an analysis of the role of mentalization in the reduction of maladaptive defense. However, we must also acknowledge some limitations since they require a careful interpretation, especially regarding the generalizability of the results. First, the study relied on self-report measures rather than on expert ratings. This approach was chosen deliberately because we were interested in the subjective experiences of the participants. However, further studies should verify our results by augmenting self-report measures with other forms of diagnostic procedures, such as expert ratings. Another limitation concerns the treatment conditions that were used in the hospitals. Even though the study does not claim to meet the standard of a randomized controlled trial, it is important to consider that we did not use a psychotherapy intervention that specifically focused on an increase in mentalization and that the participants were not compared to a placebo or non-treatment control group. Therefore, we cannot clearly anticipate how a structured and specific treatment program, such as mentalization-based treatment (5), may have further improved the results. Future studies that compare unspecific psychotherapy vs. mentalization-based treatment and placebo intervention or an intervention with patients on a waiting list are needed. Finally, inpatient therapy did not lead to a significant reduction in intermediate defense or a significant increase in adaptive defense. Therefore, the data suggest that there is no association of intermediate defense or adaptive defense with mentalization, but we cannot exclude this possibility with certainty. Since other studies have documented the possibility for changes in more mature defense mechanisms via psychotherapy (28, 69), it is advisable to verify our results in a study with long-term psychotherapy.

## Conclusion

This study is the first to empirically investigate the role of mentalization in changes in defense mechanisms over the course of rehabilitation from psychiatric disorders and during posttreatment follow-up. We detected a significant increase in mentalization and a significant reduction in maladaptive defense, psychopathology, and interpersonal problems. However, more mature defense styles did not change significantly during inpatient therapy or follow-up. Our data suggest that the reduction in maladaptive defense can be significantly predicted by an increase in mentalization both during and after inpatient therapy, whereas reductions in psychopathology and interpersonal problems appear to be less important. Mentalization appears to promote healthier ways to cope with negative stimuli, as it may reduce the prevalence of immature defense mechanisms. However, we could not find implications of the effects of mentalization on intermediate and adaptive defense mechanisms. Longer and more intense psychotherapy approaches may be necessary to foster more mature defense styles.

## Data Availability Statement

The raw data supporting the conclusions of this article will be made available by the authors, without undue reservation.

## Ethics Statement

The study was reviewed and approved by Ethikkommission des Landes Kärnten Klinikum Klagenfurt am Wörthersee, Feschnigstraße 11 A-9020 Klagenfurt a. W., Austria. The patients provided their written informed consent to participate in this study.

## Author Contributions

MH, SA, MD, PM, and KB discussed the research question and derived the hypotheses. MH, SA, and PM designed the study. MH, RG, and BS directed the study and supervised the data collection in the hospitals. Data were collected by MH and PM. MH and KB analyzed the data and discussed the results with supervision from SA. The first draft of the manuscript was written by MH. All authors contributed and agreed to the final manuscript.

## Conflict of Interest

The authors declare that the research was conducted in the absence of any commercial or financial relationships that could be construed as a potential conflict of interest.
